# Inflammation-related adverse reactions following vaccination potentially indicate a stronger immune response

**DOI:** 10.1080/22221751.2021.1891002

**Published:** 2021-03-01

**Authors:** Chun-Lan Zhuang, Zhi-Jie Lin, Zhao-Feng Bi, Ling-Xian Qiu, Fang-Fang Hu, Xiao-Hui Liu, Bi-Zhen Lin, Ying-Ying Su, Hui-Rong Pan, Tian-Ying Zhang, Shou-Jie Huang, Yue-Mei Hu, You-Lin Qiao, Feng-Cai Zhu, Ting Wu, Jun Zhang, Ning-Shao Xia

**Affiliations:** a State Key Laboratory of Molecular Vaccinology and Molecular Diagnostics, National Institute of Diagnostics and Vaccine Development in Infectious Diseases, Strait Collaborative Innovation Center of Biomedicine and Pharmaceutics, School of Public Health, Xiamen University, Xiamen, People’s Republic of China; b Xiamen Innovax Biotech CO., Ltd., Xiamen, People’s Republic of China; c Jiangsu Provincial Center for Disease Control and Prevention, Public Health research institute of Jiangsu Province, Nanjing, People’s Republic of China; d Chinese Academy of Medical Sciences/Peking Union Medical College School of Population Medicine and Public Health, Beijing, People’s Republic of China

**Keywords:** Inflammation, adverse reaction, recombinant vaccine, immune response, antibody, vaccine hesitancy

## Abstract

Concerns about vaccine safety are an important reason for vaccine hesitancy, however, limited information is available on whether common adverse reactions following vaccination affect the immune response. Data from three clinical trials of recombinant vaccines were used in this post hoc analysis to assess the correlation between inflammation-related solicited adverse reactions (ISARs, including local pain, redness, swelling or induration and systematic fever) and immune responses after vaccination. In the phase III trial of the bivalent HPV-16/18 vaccine (Cecolin®), the geometric mean concentrations (GMCs) for IgG anti-HPV-16 and -18 (*P*<0.001) were significantly higher in participants with any ISAR following vaccination than in those without an ISAR. Local pain, induration, swelling and systemic fever were significantly correlated with higher GMCs for IgG anti-HPV-16 and/or anti-HPV-18, respectively. Furthermore, the analyses of the immunogenicity bridging study of Cecolin® and the phase III trial of a hepatitis E vaccine yielded similar results. Based on these results, we built a scoring model to quantify the inflammation reactions and found that the high score of ISAR indicates the strong vaccine-induced antibody level. In conclusion, this study suggests inflammation-related adverse reactions following vaccination potentially indicate a stronger immune response.

## Introduction

Vaccines are widely recognized as one of the greatest public health successes of the last century, significantly reducing morbidity and mortality from a variety of bacterial and viral infections. However, vaccine hesitancy, especially concerns about vaccine safety or adverse reactions, impedes the implementation of vaccines such as the human papillomavirus (HPV) vaccine [1–3] and measles-containing vaccine [4,5]. Currently, some innovative candidate vaccines against coronavirus disease 2019 (COVID-19) showed high immunogenicity in their early phase clinical trials [6,7], however the accompanied high incidence of moderate to severe adverse reactions makes a shadow on people’s compliance in the future implementation. Unveiling the scientific basis of adverse reactions is urgently needed to help understand mechanism of vaccines and counter vaccine hesitancy.

The different antigens or adjuvants included in vaccines differ in their mode of action and ability to stimulate the immune system [8,9]. Regardless of the mode, inflammatory responses, which are one of the biological bases for adverse reactions, are also thought to be essential for the development of adaptive immunity. A prime example is the high incidence of febrile reactions and sufficient antibody responses in young children compared with poor immunogenicity and low reactogenicity in adults after receiving an alum-adjuvanted H5N1 whole virion-inactivated vaccine [10]. The Bacillus Calmette Guerin (BCG) scar, a permanent scar resulting from acute localized inflammation through intradermal injection, has proven to be the signal of successful vaccination [11,12].

Reactogenicity represents the physical manifestation of the inflammatory response to vaccination and can include injection-site pain, redness, swelling or induration, as well as systemic symptoms such as fever [13]. These symptoms are also the most common transient adverse reactions following clinical vaccination [14–16]. Although it is a long tale that the stronger reactivity of a vaccine usually denotes a stronger immune response [17–20], few straightforward data from large-scale human trials was reported previously.

In this study, we aimed to explore the relationship between adverse reactions that may be caused by inflammation and immune responses following vaccinations on the basis of data from clinical trials of recombinant HPV and hepatitis E virus (HEV) vaccines. Furthermore, we attempted to build a simple model to quantify adverse reactions, which can be an approach to hierarchically assess individual levels of inflammatory responses after vaccination.

## Materials and methods

### Study design and data sources

The data used in this study were from three published clinical trials of two different recombinant vaccines. We explored a primary study, a multicenter, randomized, double-blind, phase III trial of a novel *Escherichia coli*-produced bivalent HPV-16/18 vaccine (Cecolin®) involving females aged 18–45 years (NCT01735006) [21]. We further verified key findings in two other trials, including an immunogenicity bridging study of Cecolin® involving females aged 9–26 years (NCT02562508) [22] and the reactogenicity subset of a large-scale, randomized, double-blind, placebo-controlled, phase III trial of the HEV vaccine (Hecolin®) involving males and females aged 16–65 years (NCT01014845) [23]. All studies were approved by the Independent Ethics Committee (the phase III trial of Cecolin®:12-72/606, 2020-48, 2012044, IRB00001594; the immunogenicity bridging study of Cecolin®: JSJK2015-A010-02; the phase III trial of Hecolin®: no ethics committee protocol number available due to the imperfect ethical review system at the time) and undertaken according to Good Clinical Practice.

### Vaccines

Both licensed vaccines were recombinant vaccines manufactured by Xiamen Innovax, Xiamen, China, as described previously [21–24]. The bivalent HPV-16/18 vaccine (Cecolin®) is a mixture of two aluminum hydroxide adjuvant-absorbed recombinant L1 Virus Like Particles (VLPs) of HPV-16 and HPV-18 expressed in *E. coli* [25–27]. The formulation comprised 40 μg of HPV-16 and 20 μg of HPV-18 L1 VLPs suspended in 0.5 ml of buffered saline containing 208 μg of aluminum adjuvant. In the phase III trial, women in the test group were randomly vaccinated with three batches of the HPV vaccine (Lot 1: B20120404; Lot 2: B20120405; and Lot 3: B20120506), and in the immunogenicity bridging study, participants were vaccinated with the same batch of the HPV vaccine (Lot: B20141201). The HEV 239 vaccine (Hecolin®) contains 30 μg of the purified antigen adsorbed to 280 μg aluminum adjuvant suspended in 0.5 mL buffered saline [28,29].

### Safety data collection

Both vaccines were administered intramuscularly at day 0, month 1 and 6. The participants were requested to stay for at least 30 mins after each vaccination, and any adverse reactions observed were documented by the investigators. All the participants were trained to record adverse events (AEs), concomitant medications and concomitant vaccinations occurring within 1 month after each injection on diary cards, and the investigators followed the participants twice in 7 days after each vaccination by visiting households or making phone calls to ensure the integrity of records. The AE documents contained the time of occurrence, duration and severity that followed guiding principles enacted by the National Medical Products Administration (NMPA; Supplementary Table 1). Trained health care workers reviewed the returned diary cards for completeness and accuracy. Investigators with relevant qualifications would determine the causality of adverse reactions/events according to the implementation rules (Supplementary Table 2), which would be reviewed again by the Data and Safety Monitoring Board (DSMB) independent of the clinical trials to confirm the correlation between adverse reactions/events and the vaccines. The correlationship was classified as positive correlation, high probability correlation, possible correlation, possible irrelevance and irrelevance (with the first three categories considered vaccine related).

### Immunogenicity assessments

Serum samples were collected at day 0 and month 7 for all the participants to quantitatively measure IgG antibodies as described previously [21–23]. IgG antibodies against HPV-16, HPV-18 and HEV were quantified using references traceable to the WHO standards for antibodies against HPV-16 (NIBSC code 05/134), HPV-18 (NIBSC code 10/140) and HEV (NIBSC code 95/584), respectively. The lower detection limits of the assays were 3.1 IU/ml for HPV-16 antibodies, 2.0 IU/ml for HPV-18 antibodies and 0.077 Wu/ml for HEV antibodies. Antibody titers below the lower detection limit of the assay were given an arbitrary value of half the cutoff value for calculating the geometric mean concentration (GMC).

### Statistical analysis

This post hoc analysis of safety and immunogenicity was performed on participants who complied with the protocol, received 3 doses of vaccine within the requested time window, were negative for the corresponding antibody at entry, and had IgG antibody results at month 7. We assessed the relationship between inflammation-related solicited adverse reactions (ISARs) during days 0–7 postvaccination and GMC of IgG antibodies. Covariance (ANCOVA) analysis was performed to compare the GMC based on whether there was pain, induration, redness, swelling at the injection site or fever, which were generally considered to be more typical symptoms of inflammation in the immune response.

We subsequently attempted to set up a calculating method to quantify the inflammatory response based on the data of Cecolin®. Each symptom and different level of severity were given a weight coefficient (determined by GMC ratio). Each individual in the data set (DS) had a score of inflammation-related solicited adverse reactions (SI) that was defined by:

$$SI=\sum \left(W_{i,j\;}\cdot W_{s}\right)$$

where *W_i,j_* and *W_s_* are the weight coefficients of a certain symptom *i* (*i *= pain or induration or redness or swelling or fever) after dose *j* (*j*=1,2,3) and level of severity (*s*=1,2,3), respectively. Thus, each ISAR with a certain level of severity occurring after any dose would be counted in the score. ANCOVA was then also used to compare the levels of antibodies in different groups of SI.

All analyses were performed using IBM SPSS Statistics 22. A *p*-value <0.05 was considered statistically significant.

## Results

### A tentative exploration

**Figure 1.**
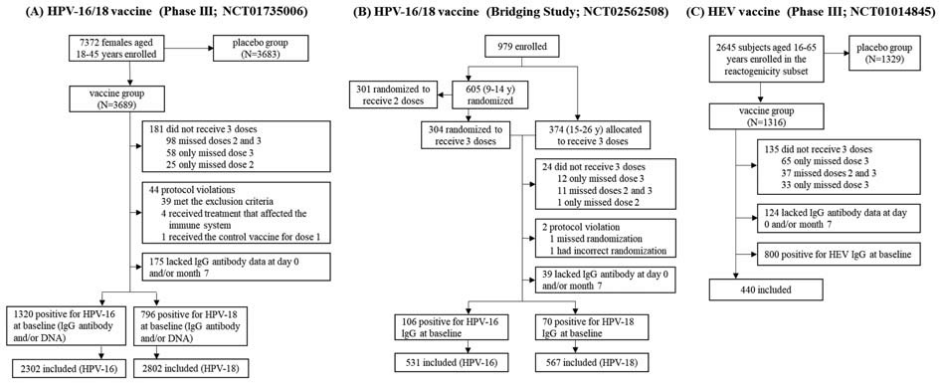
Flowchart of the studies.

We used the phase III trial of Cecolin® as our preliminary exploratory study. A total of 2302 and 2802 participants were included in the DS for HPV-16 and 18, respectively (Figure 1). Approximately 58.1% (1338/2302) and 58.6% (1642/2802) of the women in the HPV-16 and HPV-18 DS, respectively, reported at least one ISAR; among them, approximately 39.8% (532/1338) and 39.4% (647/1642) experienced ISAR after two or more injections, and most of the reactions were mild with grade 1 or grade 2.

Pain at the injection site (34.8% in the DS-HPV-16; 34.5% in the DS-HPV-18) and fever (35.2% in the DS-HPV-16; 35.6% in the DS-HPV-18) were the two most common solicited adverse reactions. On covariance analysis (Table 1), the GMCs for anti-HPV-16 and 18 IgG were significantly higher in women who had ISAR following vaccination than in those who had no ISARs (HPV16: 841.6 IU/ml [95% CI 810.0–874.4] vs 724.5 IU/ml [95% CI 692.6–757.9], *P_16 _*< 0.001; HPV18: 279.5 IU/ml [95% CI 269.4–290.0] vs 252.2 IU/ml [95% CI 241.4–263.5], *P_18 _*< 0.001). Also, the incidence of ISAR was higher in women with higher titer of antibodies after vaccination (Supplementary Figure 1). A relatively larger proportion of women aged 18–26 years had ISARs than women aged 27–45 years (63.6% vs 53.1%, Table 1), accompanied by higher antibody GMCs in women aged 18–26 years than in women aged 27–45 years. The limited available immuno-persistence data showed that the correlation of ISAR incidence with long-term IgG antibody levels (at month 18, 30 and 42m) showed similar trends, although significant difference was presented only in the DS for HPV-18 (Supplementary Table 3).

**Table 1.** Antibody levels of HPV-16 and -18 IgG in the presence of different ISAR (a phase III trial of Cecolin®).

ISAR	HPV-16 (*N*=2302)					HPV-18 (*N*=2802)			
	*n* (%)	median age (interquartile range)	IgG GMC(95% CI)	*P*		*n* (%)	median age (interquartile range)	IgG GMC(95% CI)	*P*
any ISAR									
No	964 (41.9%)	30.5 (25,39)	724.5 (692.6,757.9)	<0.001		1160 (41.4%)	30 (25,39)	252.2 (241.4,263.5)	<0.001
Yes	1338 (58.1%)	26 (24,35)	841.6 (810.0,874.4)			1642 (58.6%)	26 (24,35)	279.5 (269.4,290.0)	
18–26 y									
No	400 (36.4%)	24 (23,25)	858.8 (800.6,921.2)	0.057		489 (36.1%)	24 (23,25)	292.9 (273.8,313.4)	0.415
Yes	700 (63.6%)	24 (23,25)	935.4 (887.1,986.3)			866 (63.9%)	24 (23,25)	303.4 (288.4,319.2)	
27–45 y									
No	564 (46.9%)	38 (33,42)	642.2 (606.5,680.0)	<0.001		671 (46.4%)	38 (33,41)	226.2 (213.8,239.3)	0.002
Yes	638 (53.1%)	35 (31,40)	749.4 (710.2,790.8)			776 (53.6%)	36 (31,40)	255.1 (242.1,268.8)	
Maximum severity									
grade 1 or 2	1317 (98.4%)	26 (24,35)	837.4 (805.6,870.5)	0.047		1612 (98.2%)	26 (24,35)	278.0 (267.8,288.7)	0.037
grade 3	21 (1.6%)	26 (23,33)	1145.5 (842.7,1557.2)			30 (1.8%)	25 (23,33)	373.8 (283.8,492.3)	
The number of injections resulting in ISARs									
1	806 (60.2%)	27 (24,36)	809.7 (770.6,850.8)	0.023		995 (60.6%)	27 (24,36)	275.3 (262.4,288.8)	0.524
2	405 (30.3%)	26 (24,33)	872.3 (813.5,935.4)			497 (30.3%)	26 (24,34)	283.5 (264.9,303.4)	
3	127 (9.5%)	26 (24,31)	958.8 (846.4,1086.2)			150 (9.1%)	26 (24,31)	295.0 (260.8,333.8)	
Pain									
No	1501 (65.2%)	28 (24,38)	766.2 (739.0,794.5)	0.004		1835 (65.5%)	28 (24,38)	258.6 (249.8,267.8)	0.001
Yes	801 (34.8%)	26 (24,35)	837.7 (797.1,880.3)			967 (34.5%)	26 (24,35)	286.4 (273.0,300.5)	
Induration									
No	2137 (92.8%)	27 (24,37)	783.9 (760.4,808.1)	0.047		2594 (92.6%)	27 (24,37)	266.1 (258.4,274.0)	0.097
Yes	165 (7.2%)	26 (24,36)	879.5 (788.3,981.3)			208 (7.4%)	26 (24,36)	291.5 (262.8,323.2)	
Redness									
No	2197 (95.4%)	27 (24,37)	787.0 (763.8,811.0)	0.193		2679 (95.6%)	27 (24,37)	266.8 (259.2,274.6)	0.172
Yes	105 (4.6%)	29 (25,38)	864.0 (753.2,991.2)			123 (4.4%)	28 (25,38)	293.6 (256.6,335.9)	
Swelling									
No	2193 (95.3%)	27 (24,37)	782.6 (759.4,806.4)	0.003		2675 (95.5%)	27 (24,37)	264.8 (257.3,272.6)	<0.001
Yes	109 (4.7%)	29 (25,39)	965.6 (844.0,1104.7)			127 (4.5%)	27 (24,39)	340.8 (298.6,388.9)	
Fever									
No	1492 (64.8%)	29 (25,39)	761.1 (734.0,789.3)	0.001		1804 (64.4%)	29 (25,38)	264.3 (255.2,273.8)	0.212
Yes	810 (35.2%)	26 (24,34)	847.3 (806.5,890.1)			998 (35.6%)	26 (24,34)	274.4 (261.8,287.7)	

ISAR, inflammation-related solicited adverse reaction; *N*, the total sample size; *n*, the number of participants; CI, confidence interval; GMC, geometric mean concentration.

Higher GMCs for anti-HPV-16 and 18 IgG were recorded in women who experienced grade 3 ISAR, and lower GMCs were recorded in those who experienced grade 1 or 2 ISAR (HPV16: 1145.5 IU/ml [95% CI 842.7–1557.2] vs 837.4 IU/ml [95% CI 805.6–870.5], *P_16 _*= 0.047; HPV18: 373.8 IU/ml [95% CI 283.8–492.3] vs 278.0 IU/ml [95% CI 267.8–288.7], *P_18 _*= 0.037). With the increasing number of injections resulting in ISARs, there was an upward trend in GMCs, although a significant difference was only noted in GMCs for anti-HPV-16 (*P_16_* = 0.023). When ISAR was subdivided into five symptoms, we found that there was a trend towards higher GMCs in women with any one of the symptoms overall. Significant differences were observed in both HPV types IgG between women with and without pain (*P_16_ *= 0.004; *P_18_* = 0.001) or swelling (*P_16_* = 0.003; *P_18_* < 0.001) at the injection site, as well as anti-HPV-16 IgG between women with and without induration at the injection site (*P_16_* = 0.047) or fever (*P_16_* = 0.001).

### Validation and further analysis

Similar findings with significantly higher GMCs in individuals who had ISAR following vaccination were revealed by the analysis of the bridging study of Cecolin® (Table 2, Supplementary Figure 1) and the phase III trial of the HEV vaccine (Table 3, Supplementary Figure 1). In the DS of the bridging study, GMCs of IgG against HPV-16 were significantly higher in girls or young women who experienced ISARs versus no ISARs (2537.0 IU/ml [95% CI 2338.0–2752.8] vs 2057.3 IU/ml [95% CI 1882.3–2248.5], *P_16 _*= 0.001). Men and women with ISARs had significantly higher GMCs for anti-HEV IgG than those without ISARs in the DS of the phase III trial of HEV vaccine (21.38 WU/ml [95% CI 18.86–24.23] vs 16.74 WU/ml [95% CI 15.31–18.29], *P_HEV_ *= 0.002). No obvious increasing trend was observed on GMCs in increasing severity or number of injections resulting in ISARs. Significant differences were observed in GMCs for anti-HPV-16 and anti-HEV IgG between subjects with fever and those without fever (HPV16: 2613.9 IU/ml [95% CI 2357.4–2898.3] vs 2159.8 IU/ml [95% CI 2005.2–2326.3], *P_16 _*= 0.003; HEV: 21.06 WU/ml [95% CI 18.09–24.52] vs 17.38 WU/ml [95% CI 15.99–18.89], *P_HEV_ *= 0.030).

**Table 2.** Antibody levels of HPV-16 and -18 IgG in the presence of different ISAR (a bridging study of Cecolin®).

ISAR	HPV-16 (*N*=531)					HPV-18 (*N*=567)			
	*n*(%)	median age (Interquartile range)	IgG GMC(95%CI)	*P*		*n*(%)	median age (Interquartile range)	IgG GMC(95%CI)	*P*
any ISAR									
No	243 (45.8%)	15 (12,21)	2057.3 (1882.3,2248.5)	0.001		261 (46.0%)	15 (12,21)	501.8 (454.4,554.1)	0.094
Yes	288 (54.2%)	15 (12,19)	2537.0 (2338.0,2752.8)			306 (54.0%)	15 (11,19)	563.2 (513.9,617.3)	
9-16y									
No	163 (44.5%)	13 (11,15)	2508.9 (2269.1,2773.9)	0.017		172 (44.2%)	13 (11,15)	655.8 (583.6,736.9)	0.981
Yes	203 (55.5%)	13 (11,15)	2957.8 (2703.2,3236.3)			217 (55.8%)	13 (10,15)	657.0 (592.2,728.9)	
18-26y									
No	80 (48.5%)	23 (21,25)	1373.1(1184.9,1591.2)	0.019		89 (50%)	23 (21,25)	299.1 (257.9,346.9)	0.016
Yes	85 (51.5%)	22 (20,25)	1758.4(1524.1,2028.9)			89 (50%)	23 (20,25)	386.8 (333.5,448.6)	
Maximum severity									
grade 1 or 2	282 (97.9%)	15 (12,19)	2546.5 (2338.3,2773.3)	0.547		300 (98.0%)	15 (12,19)	556.8 (508.7,609.4)	0.075
grade 3	6 (2.1%)	12.5 (9,16)	2124.4 (1183.7,3812.7)			6 (2.0%)	13.5 (9,23)	1000.4 (528.2,1894.9)	
The number of injections occurring ISAR									
1	186 (64.6%)	14.5 (12,20)	2488.2 (2239.7,2764.3)	0.797		197 (64.4%)	14 (12,20)	560.3 (500.8,626.8)	0.904
2	84 (29.2%)	15 (11,17.5)	2603.3 (2226.1,3044.5)			91 (29.7%)	15 (11,18)	560.4 (475.1,661.0)	
3	18 (6.3%)	15 (12,19)	2747.9 (1959.4,3853.7)			18 (5.9%)	15 (12,19)	611.4 (421.8,886.2)	
Pain									
No	395 (74.4%)	15 (11,19)	2281.3 (2126.1,2447.8)	0.570		415 (73.2%)	15 (11,19.5)	531.0 (490.7,574.6)	0.783
Yes	136 (25.6%)	15 (13,20.5)	2375.0 (2106.3,2678.0)			152 (26.8%)	16 (13,21)	542.5 (476.2,618.0)	
Induration									
No	503 (94.7%)	15 (12,19.5)	2298.8 (2159.6,2446.9)	0.714		535 (94.4%)	15 (11,20)	535.2 (499.3,573.8)	0.790
Yes	28 (5.3%)	16 (14,22)	2418.5 (1856.0,3151.3)			32 (5.6%)	17 (14.5,23)	514.4 (387.1,683.4)	
Redness									
No	508 (95.7%)	15 (12,20)	2290.0 (2152.1,2436.7)	0.322		543 (95.8%)	15 (12,20)	531.7 (496.3,569.7)	0.545
Yes	23 (4.3%)	16 (13.5,21.5)	2662.1 (1988.3,3564.1)			24 (4.2%)	16.5 (14.5,22.5)	589.5 (424.7,818.4)	
Swelling									
No	502 (94.5%)	15 (12,20)	2276.5 (2138.9,2423.0)	0.095		536 (94.5%)	15 (12,20)	528.1 (492.8,566.0)	0.178
Yes	29 (5.5%)	16 (15,21)	2857.8 (2204.8,3704.3)			31 (5.5%)	16 (14.5,21.5)	647.4 (485.3,863.7)	
Fever									
No	350 (65.9%)	15 (12,21)	2159.8 (2005.2,2326.3)	0.003		372 (65.6%)	15 (12,21)	512.7 (471.8,557.2)	0.102
Yes	181 (34.1%)	14 (11,17)	2613.9 (2357.4,2898.3)			195 (34.4%)	14 (11,17)	577.2 (514.6,647.4)	

ISAR, inflammation-related solicited adverse reaction; *N*, the total sample size; *n*, the number of participants; CI, confidence interval; GMC, geometric mean concentration.

**Table 3.** Antibody levels of HEV in the presence of different ISAR (a phase III trial of HEV vaccine).

ISAR	*n*(%)	Gender ratio* (male: female)	Median age (interquartile range)	Anti-HEV IgG GMC(95%CI)	*P*
Any ISAR					
No	293 (66.6%)	103: 190	42 (35,52)	16.74 (15.31,18.29)	0.002
Yes	147 (33.4%)	27: 120	41 (36,48.5)	21.38 (18.86,24.23)	
16-40y					
No	129 (66.2%)	43: 86	32 (27,37)	18.35 (16.11,20.91)	0.209
Yes	66 (33.8%)	13: 53	35 (28,38)	21.18 (17.65,25.43)	
41-65y					
No	164 (66.9%)	60: 104	51 (47,57)	15.57(13.79,17.58)	0.003
Yes	81 (33.1%)	14: 67	48 (43,53)	21.54(18.12,25.59)	
Maximum severity					
grade 1 or 2	145 (98.6%)	27: 118	41 (36,49)	21.76 (19.44,24.36)	-
grade 3	2 (1.4%)	0: 2	33.5 (24,43)	5.85 (2.24,15.28)	
The number of injections occurring ISAR					
1	112 (76.2%)	23: 89	41.5 (36,49)	20.73 (18.18,23.65)	0.643
2	27 (18.4%)	3: 24	43 (36,48)	23.59 (18.05,30.85)	
3	8 (5.4%)	1: 7	38 (36,39.5)	23.51 (14.37,38.47)	
Pain					
No	392 (89.1%)	121: 271	42 (35,52)	17.80 (16.47,19.23)	0.117
Yes	48 (10.9%)	9: 39	41 (35.5,48)	21.46 (17.20,26.78)	
Induration					
No	434 (98.6%)	128: 306	42 (35,51)	18.09 (16.80,19.47)	0.356
Yes	6 (1.4%)	2: 4	39 (35,49)	24.34 (13.00,45.57)	
Redness					
No	435 (98.9%)	130: 305	42 (35,51)	18.09 (16.81,19.47)	0.317
Yes	5 (1.1%)	0: 5	44 (40,54)	25.73 (12.95,51.14)	
Swelling					
No	427 (97.0%)	130:297	42 (35,51)	17.96 (16.68,19.35)	0.089
Yes	13 (3.0%)	0: 13	40 (38,43)	26.13 (17.08,39.97)	
Fever					
No	339 (77.0%)	109: 230	42 (35,51)	17.38 (15.99,18.89)	0.030
Yes	101 (23.0%)	21: 80	41 (37,51)	21.06 (18.09,24.52)	

ISAR, inflammation-related solicited adverse reaction; *N*, the total sample size; *n*, the number of participants; CI, confidence interval; GMC, geometric mean concentration; NA, not applicable.

* There was no significant difference in antibody levels between the females and the males (*P*=0.147).

### Quantitative model

**Figure 2.**
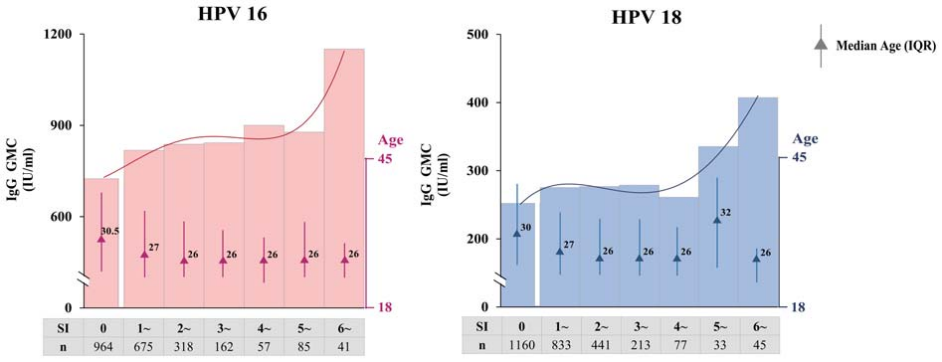
HPV-16 and HPV-18 antibody levels at different SI values. According to the model, in the phase III trial of Cecolin®, the SI of subjects who never experienced an ISAR following vaccination was 0, while subjects with any ISAR had a SI between 1.00 and 12.44 in the DS of HPV-16 and 1.00–11.34 in the DS of HPV-18. All subjects were grouped based on their SI; the bar represents the GMC of HPV antibodies. Quadrinomial fitting curves are presented. GMC: geometric mean concentration.

Based on the results from the analysis of the phase III trial and the bridging study of Cecolin®, we used the GMC ratio as the weight coefficients (Table 4) and obtained a SI at the individual level. According to the model, the SI of subjects who never experienced an ISAR following vaccination was 0, while subjects with any ISAR had a SI between 1.00 and 12.44 in the DS of HPV-16 and 1.00 and 11.34 in the DS of HPV-18. As shown in Figure 2, there were nonlinear curves between the SI and GMCs, and the GMCs tended to be higher as the SI increased to a certain level.

**Table 4.** Weight coefficient*.

ISAR	HPV16	HPV18
Severity (Ws)		
1 or 2	1.16	1.10
3	1.58	1.48
Symptoms (Wi)		
Pain	1.09	1.11
Induration	1.12	1.10
Redness	1.10	1.10
Swelling	1.23	1.29
Fever	1.11	1.04

ISAR: inflammation-related solicited adverse reaction; Ws: the weight coefficient of severity; Wi: the weight coefficient of symptoms.

* Weight coefficients are derived from the ratio of geometric mean concentrations (GMCs) for antibodies. For example, the weight coefficient of “pain” is calculated by the ratio of GMCs for antibodies of vaccinees with pain to that of vaccinees without pain.

## Discussion

To our knowledge, this is the first study to systematically analyze the correlation between inflammation-related solicited adverse reactions and specific immune responses induced by vaccines in humans with a large sample size. In the phase III trial of Cecolin®, we observed significantly higher GMCs for anti-HPV-16 and -18 in vaccinees who had ISARs compared with those without ISARs. For each of the five ISAR symptoms, there was a trend towards higher GMCs for anti-HPV-16 and/or -18 in women with pain, induration, redness, or swelling at the injection site or fever compared with those without the relative symptom, although some differences were not significant. The findings were validated by data from another two clinical trials of recombinant vaccines.

The prerequisite for eliciting immune responses is to provide sufficient “danger signals” by triggering an inflammatory reaction mediated by cells of the innate immune system [30–33], which followed a complex series of innate immune events, such as phagocytosis, release of inflammatory mediators including chemokines and cytokines, activation of complement, liberation of vasodilators and cellular recruitment [30–34]. Vasodilators and the chemokine gradient facilitate cell recruitment from blood but also lead to the development of redness and swelling at the injection site [13]. When vaccines, especially adjuvanted vaccines, cannot be absorbed in a timely fashion and thus remain at the injection site, induration at the injection site can occur due to a prolonged inflammatory response [35]. Induration is a special manifestation of acute inflammation due to the local extravasation and attraction of immune cells in response to long-living “foreign matter”. Besides, immune cells may initiate the sensitization of peripheral nociceptors by releasing soluble factors, such as cytokines, prostaglandins or ATP, and interactions with neurotransmitters and their receptors [36]. Recent studies [37,38] have shown an important role of the sensory nervous system in mechanisms controlling antigen-specific antibody responses. In our study, pain at the injection site is also considered to be highly correlated with higher antibody levels following HPV vaccination.

Meanwhile, mediators and products of inflammation in the circulation may affect other body systems or organs to cause systemic adverse reactions such as fever [13]. The fact that fever promotes the immune response has been validated by numerous studies [17–19]. However, as a typical manifestation of inflammatory responses, fever can be caused by a number of complex factors and can also be easily treated with physical or pharmaceutical interventions, which may be some of the reasons that make it more difficult to find a strong correlation between fever and antibodies induced by vaccines in the real world. A meta-analysis concluded that while prophylactic antipyretics significantly reduced injection-site and systemic symptoms in children after vaccination, their use was associated with reduced antibody responses to most vaccines. However, whether antipyretic interventions before or after vaccination do more harm than good cannot be answered before more clinical evidence is obtained.

Table 1 shows that the median age of people with ISARs was younger than those without ISARs, a relatively larger proportion of women aged 18–26 years had ISARs than women aged 27–45 years, and the older the age, the lower the incidence of ISARs and the weaker the immune response. Undoubtedly, age is an important factor influencing reactogenicity in light of physiological functions of the immune and nervous systems that evolve throughout life, including the susceptibility to adverse reactions to vaccination. Fewer reported ISARs in relatively older people is possibly due to higher tolerance to pain or the waning of innate immune defense mechanisms [39], which was supported by the results of a study [40] that older people display lower systemic levels of IL-6, IL-10 and C-reactive protein (CRP) after vaccination. At the same time, it has become evident that young people tend to gain higher antibody levels after vaccination [41]. Hence, the factor of age, to a certain extent, explains the inevitable connection between immunogenicity and adverse reactions from the perspective of the individual characteristics. Moreover, according to the results of age-stratified analysis shown in Tables 1–3, it seems that the positive correlations between ISAR and IgG antibodies are more pronounced in the older age groups than that in the younger age groups, which suggest that age might also be a potential influencing factor on the correlation of reactogenicity with adaptive immune response.

Equally important, as immunostimulants, adjuvants enhance the immune response to the antigen and usually increase reactogenicity [42,43]. The HPV-16/18 bivalent vaccine Cervarix® (GlaxoSmithKline, GSK) formulated by virus-like particles of the L1 protein and the Adjuvant System 04 (AS04), which is a combination of monophosphoryl lipid A (MPL) and aluminum salts, has shown good immunogenicity in clinical studies [44–46]. MPL is a specific agonist of TLR4 (Toll-like receptor), which is a kind of innate receptor (pattern recognition receptor, PRR) that can recognize conserved motifs expressed by microbes. Stimulation of TLR4 mimics the existence of “danger” [47]. Previous studies [48] on the mechanism of action of AS04 provided evidence that MPL enhances humoral and cell-mediated responses by rapidly triggering a local and transient cytokine response that leads to an increased activation of APCs and induces an improved presentation of antigen to CD4+ T cells, which can explain the higher levels of cytokines at the injection site [49] and the higher immunogenicity [50] of the AS04-adjuvanted vaccine compared with aluminum salt-based vaccines. However, a comparative study [51,52] on the immunogenicity and safety between Cervarix® and Gardasil® (HPV6/11/16/18: MSD, USA) suggested a higher incidence of solicited symptoms (injection-site reactions being most common) following the vaccination of Cervarix®. The similar phenomenon is common in studies on other vaccines, for instance, glycoprotein E adjuvanted with AS01_B_ for Shingrix® leads to high reactogenicity than that without adjuvant while it greatly enhances the cellular and humoral immunogenicity [53], and the results of the phase 1/2 clinical trial of a recombinant COVID-19 vaccine showed that the formulation with Matrix-M1 induced much higher neutralizing antibody than that without adjuvant accompanied with more ISARs [54]. All these data further illustrated that inflammation induced by vaccines and their clinical manifestations may enhance the production of specific antibodies.

It is interesting to note that the severity and the number of injections resulting in ISARs also appear to be influencing factors for the levels of antibodies, according to the results of the phase III trial of Cecolin®. To our knowledge, such an impact of the severity and frequency of adverse reactions on antibody production has not been documented before. However, this correlation is only shown significance in HPV-16 specific antibody response of the phase III trial. The possible reasons are as follows: (1) The effect of the number of injections on the immune response is indeed very small or even negligible, it is due to other unknown confounding factors that cause a significant correlation in HPV-16 specific antibody response of the phase III trial. (2) The quantitative results of immunogenicity induced by different antigens or different reagents are incomparable. The discrimination between different antibody levels induced by the vaccine’s components of HPV-18 might be inferior to that of HPV-16, which could reduce the power of statistical analysis. The significant correlation is hard to show in overall lower antibody levels. (3) The limited sample size of the bridging study of Cecolin® and the phase III trial of HEV vaccine.

We also built a scoring model to assess ISARs following vaccination. The parameters of the model are derived from the ratio of GMCs for antibodies, considering five typical symptoms of ISARs and the levels of severity of each ISAR. SI shows a similar exponential relation to GMCs for anti-HPV-16 and 18 IgG in the phase III trial of Cecolin®. This indicates that there are differences in the levels of antibodies between vaccinees with a SI below 6 and those with a SI of 6 or higher. However, the reason for the curve relationship is hard to explain because of the limited sample size of the high SI group in the present study. Notwithstanding that the quantitative model presented here is of limited clinical value, more important is the assumption of a quantifiable inflammatory response following vaccination. Although we know that an effective adaptive immune response requires a certain level of inflammation to be triggered [55], how to build a proper model and how to choose the most reliable symptoms or indicators to quantify the inflammatory level warrant further study.

Vaccine-induced immune effectors are essentially antibodies produced by B lymphocytes that can bind specifically to a toxin or a pathogen. The above mentioned improved innate immune system accompanied with more severe ISAR would promote the antigen presentation by antigen presenting cell (APC) and stimulate T cell-dependent B cell differentiate into antibody-secreting cells and memory B cells. From the results in Supplementary Table 3, we also found that the long-term IgG antibody against HPV-18 is significantly correlated with ISAR, which may be largely related to the role of memory B cells.

Currently, vaccines have largely been developed empirically, with limited knowledge of how exactly they activate the immune system [8], let alone the extent to which adverse reactions are caused by the reactogenicity of vaccines. Since the beneficial effects of vaccines are a result of changes in the immune system, it would not be surprising if some of the adverse reactions were also. Thus, more studies are needed to learn more about adverse reactions following vaccination. One approach is to observe and study adverse reactions during clinical trials to explore which adverse reactions are predictive of improved immunization outcomes. Indeed, the development of vaccines should appropriately focus on this aspect and not only on the avoidance of adverse reactions. There is uncertainty regarding whether our findings on ISARs and immunogenicity translate into clinically meaningful effects. However, these results can change people’s perception of adverse reactions following vaccination and may be a powerful weapon in the fight against vaccine hesitancy.

The limitations of this study includes that only data of recombinant, adjuvanted vaccines are analyzed. The extent to which antigens and adjuvants contribute to reactogenicity in addition to other baseline physiological characteritics (such as body mass index, circadian cycle, psychological stress, etc) needs to be further confirmed and also through similar studies on other types of vaccines (live/nonlive/nonadjuvanted). And both the HPV (Cecolin®) and HEV (Hecolin®) vaccines investigated in this study showed efficacy of 100% in the phase 3 clinical trial against clinical endpoints, thus they are not the proper data sets for analyzing the relationship between ISAR and protection, the correlation between ISARs and the actual efficacy of vaccines is worthy of further study in the future and has practical significance.

In conclusion, this study suggests inflammation-related adverse reactions following vaccination potentially indicate a stronger immune response. However, because of incomplete knowledge about the biological mechanisms of vaccine-induced injury and the immune response process, the clinical relevance of these immunological findings warrants further assessment.

## Supplementary Material

Supplementary_material-EMI-20210120.docx

## Data Availability

The data that support the findings of this study are available from the corresponding author upon reasonable request.
