# Supplementary material for: Inflammation-related adverse reactions following vaccination potentially indicate a stronger immune response
Source: Emerg Microbes Infect. 2021 Mar 1;10(1):365–75. doi: 10.1080/22221751.2021.1891002 (PMC7928063; doi:10.1080/22221751.2021.1891002)
Supplement: Supplementary_material-EMI-20210120.docx [file TEMI_A_1891002_SM1594.docx]

**Supplementary Material**

## Supplementary Table 1 The grading principles for ISARs.

| Local adverse reaction | Mild (grade 1) | Moderate (grade 2) | Severe (grade 3) | Critical (grade 4) |
| --- | --- | --- | --- | --- |
| Pain | has no effect on activities | has effects on activities or multiple use of non-narcotic pain relievers | has effects on activities or multiple use of narcotic pain relievers | emergency or hospitalization |
| Induration | <15 mm | 15～30 mm | >30 mm | gangrene or dermatitis exfoliative |
| Redness | <15 mm | 15～30 mm | >30 mm | gangrene or dermatitis exfoliative |
| Swelling | <15 mm and has no effect on activities | 15～30 mm or has effects on activities | >30 mm or has restrictions on the daily activities | gangrene |
| Fever  (axillary's temperature) | 37.1～37.5℃ | 37.6～39.0℃ | >39.0℃ | - |

The three vaccine clinical trials mentioned in the article all followed *guideline for the grading of adverse reactions in clinical trials of prophylactic vaccines* to observe the adverse reactions after vaccination. The guideline was enacted by National Medical Products Administration (NMPA) in December, 2005. The grading principles for the inflammation-related solicited adverse reactions (ISARs) mentioned in this study are shown in Supplementary Table 1.

## Supplementary Table 2 Correlation judgment implementation rules.

| Correlation | Judgment implementation rules |
| --- | --- |
| Deterministic correlation | 1. Local adverse reactions within 7 days following vaccination. |
|  | 1. Abnormal body temperature within 30 minutes following vaccination. |
|  | 1. Systemic solicited adverse reactions following vaccination within 30 minutes, ruling out other related adverse events such as upper respiratory infection and gastroenteritis. |
| High probability correlation | 1. Abnormal body temperature or systemic solicited adverse reactions within 30 minutes to 7 days following vaccination, ruling out other related adverse events. |
|  | 1. Local adverse reactions after 7 days of vaccination. |
| Possible correlation | 1. Abnormal body temperature or systemic solicited adverse reactions within 7 days following vaccination, accompanying other related adverse events. |
| Possible irrelevance | 1. Unsolicited adverse reactions within 7 days following vaccination, ruling out other related adverse events. |
|  | 1. Abnormal body temperature or systemic solicited adverse reactions after 7 days of vaccination, ruling out other related adverse events. |
| Irrelevance | 1. Abnormal body temperature or systemic solicited adverse reactions after 7 days of vaccination, accompanying other related adverse events. |
|  | 1. Unsolicited adverse reactions within 7 days following vaccination, accompanying other related adverse events. |
|  | 1. Unsolicited adverse reactions after 7 days of vaccination. |
|  | 1. Other adverse events that have been clearly diagnosed. |

In vaccine clinical trials, the sponsors and the principal investigators developed the implementation standards shown in Supplementary Table 2 according to the relevant law and regulation, and the principles of the protocol of the trials. This implementation rule guided investigators in determining the association between adverse events and vaccination. In order to ensure the accuracy of the judgment results and the comparability of the judgment results of each study site, the *Data and Safety Monitoring Board* (DSMB) experts would review and finally confirm the judgment results after the preliminary judgment by investigators.

## Supplementary Table 3 The durability of antibody to HPV-16 and -18 IgG in the presence of different ISAR (a phase III trial of Cecolin^®^)

| ISAR | HPV-16 | | |  | HPV-18 | | |
| --- | --- | --- | --- | --- | --- | --- | --- |
|  | n(%) | IgG GMC(95%CI) | ***P*** |  | n(%) | IgG GMC(95%CI) | ***P*** |
| 18m |  |  |  |  |  |  |  |
| No | 192 (46.4%) | 130.3 (116.7, 145.4) | 0.265 |  | 233 (46.0%) | 31.9 (28.4, 35.7) | 0.024 |
| Yes | 222 (53.6%) | 141.8 (128.1, 157.0) |  |  | 273 (54.0%) | 38.1 (34.3, 42.4) |  |
| 30m |  |  |  |  |  |  |  |
| No | 192 (45.7%) | 105.8 (94.1, 118.9) | 0.369 |  | 233 (45.1%) | 21.6 (19.2, 24.4) | 0.001 |
| Yes | 228 (54.3%) | 113.7 (102.2, 126.6) |  |  | 284 (54.9%) | 28.2 (25.3, 31.4) |  |
| 42m |  |  |  |  |  |  |  |
| No | 188 (46.5%) | 90.5 (80.7, 101.5) | 0.383 |  | 226 (45.3%) | 19.4 (17.2, 21.8) | 0.009 |
| Yes | 216 (53.5%) | 97.0 (87.2, 108.0) |  |  | 273 (54.7%) | 23.9 (21.5, 26.5) |  |

ISAR, inflammation-related solicited adverse reaction; n, the number of participants; CI, confidence interval; GMC, geometric mean concentration.


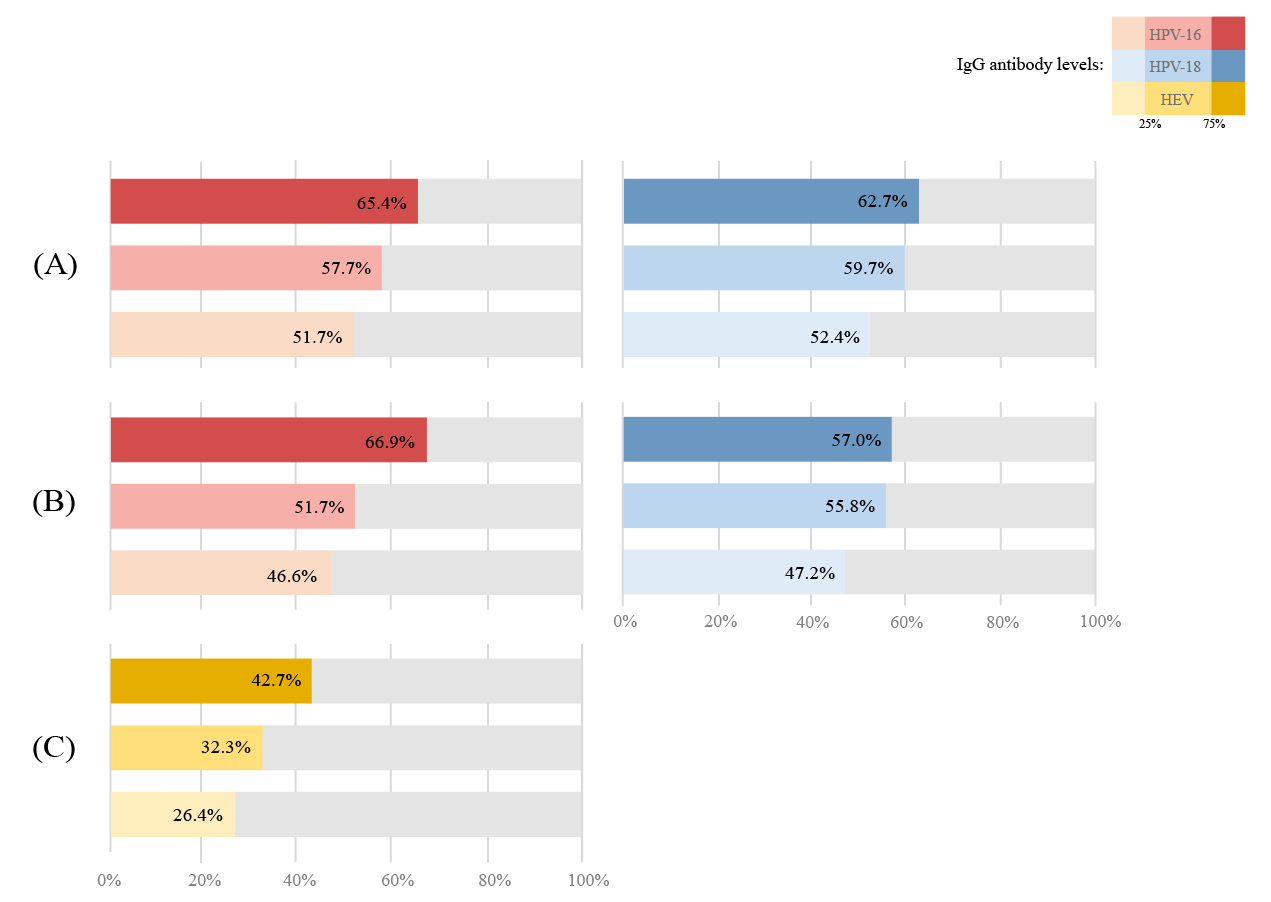
 **Supplementary fig.1 ISAR% of vaccinees with different IgG antibody levels.**

(A) The phase III trial of Cecolin^®^ (NCT01735006); (B) The immunogenicity bridging study of Cecolin^®^ (NCT02562508); (C) The phase III trial of Hecolin^®^ (NCT01014845) Vaccinees in the three trials were stratified according to quartile of overall IgG antibody level: 1) Rank-1, the IgG antibody value is below the lower quartile (25%) of the total sample in the dataset; 2) Rank-2, the IgG antibody value is between the lower and upper quartiles (25-75%) of the total sample in the dataset; 3) Rank-3, the IgG antibody value is above the upper quartile (75%) of the total sample in the dataset. The incidence of ISAR of different ranks is indicated by bar graphs with different colors (see legend).
